# Why Are So Many Bird Flowers Red?

**DOI:** 10.1371/journal.pbio.0020350

**Published:** 2004-10-12

**Authors:** Miguel A Rodríguez-Gironés, Luis Santamaría

## Abstract

Are bird-pollinated flowers red because bees - which might rob the flower of its nectar - cannot easily detect them, or might it be because of more subtle evolutionary trade-offs?

Most bird-pollinated flowers are both red and rich in nectar. The traditional explanation for this association is that, since red is inconspicuous to bees, it evolved to prevent bees from depleting the nectar of bird-pollinated flowers without effecting pollination. But bees can see, and they actually visit red flowers. So why are most bird-pollinated flowers red? To help answer this question, we need to consider how the outcomes of foraging decisions are affected by the community in which individuals live, and by the foraging options of other individuals.

## The Mystery

Plants face a trade-off between attracting pollinators and remaining hidden from flower parasites (such as nectar robbers and seed predators). Consequently, there is often strong selection pressure for highly specific communication channels that can advertise the presence of their flowers to effective pollinators but not to other individuals. Many aspects of pollinator syndromes are best understood in these terms (Proctor et al. 1996). For example, flowers that are pollinated by birds—bird flowers—produce nectar at much higher rates than those pollinated by bees (Stiles 1981). If a bee is attracted to such a flower, it might sometimes remove nectar and pollen without providing an outcrossing service (i.e., bringing pollen from a different plant of the same species) to the flower. Therefore, bird-pollinated flowers should advertise their presence to birds, but not to bees. Following this line of reasoning, Peter Raven (1972) suggested more than thirty years ago that bird-pollinated flowers were predominantly red because ‘red is the only color of the spectrum that is at once inconspicuous to most insects and also an excellent “signal” of a high caloric reward for birds’. Raven's interpretation of inconspicuousness was soon transformed into invisibility; it was assumed that bees did not visit red flowers because they couldn't detect them (Proctor et al. 1996; Vogel 1996).

**Figure 1:**
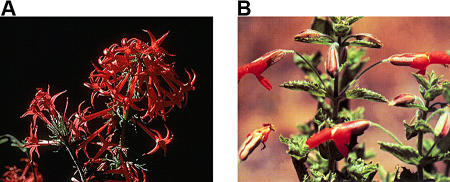
Typical Bird-Pollinated Flowers (A) Scarlet gilia Ipomopsis aggregata. Image courtesy of Clarence A. Rechenthin at US Department of Agriculture–National Resources Conservation Service PLANTS Database. (B) Scarlet monkeyflower, Mimulus cardinalis. Image by William & Wilma Follette at US Department of Agriculture–National Resources Conservation Service PLANTS Database (USDA NRCS 1992).

**Figure 2:**
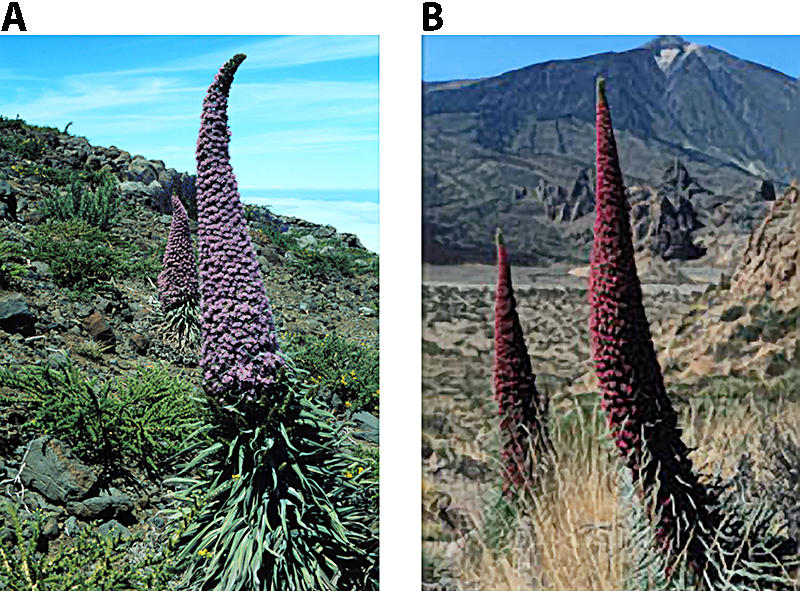
The Two Subspecies of Echium wildpretii (A) The pink flowers of E. wildpretii trichosiphon are pollinated by insects. (B) The red flowers of E. wildpretii wildpretii are pollinated by generalist native birds, unless birds are driven away by large densities of bees. Photos courtesy of Alfredo Valido.

However, this interpretation no longer holds. Chittka and Waser (1997) have shown that red flowers are not actually invisible to bees. Indeed, typical bird flowers with no UV reflectance, such as the scarlet gilia (Ipomopsis aggregata) and the scarlet monkeyflower (Mimulus cardinalis) (Figure 1), are routinely visited and exploited by different bee species (reviewed by Chittka and Waser 1997). Moreover, when bees are extremely abundant, they can drive birds away from red flowers. Echium wildpretii, an endemic of the Canary Islands, presents an entomophylous (‘insect-loving’) and an ornithophyllous (‘bird-loving’) subspecies (Figure 2) that differ in flower colour: E. wildpretii trichosiphon, endemic to La Palma Island, has entomophylous, pink flowers, whereas E. wildpretii wildpretii, endemic to Tenerife Island, has ornithophyllous, red flowers, pollinated by generalist native birds and insects. E. wildpretii wildpretii is pollinated predominantly by birds early in the season until introduced honeybees (Apis mellifera, which have increased enormously in number because of apiculture) deplete the nectar and displace nectar-feeding birds (Valido et al. 2002).

So if red flowers are not invisible to bees, why are most bird-pollinated flowers red? Perhaps birds are particularly apt at detecting red objects (Chittka and Waser 1997)? Again, this is not strictly true. Although all birds detect red objects and some birds do have their greatest spectral sensitivity and finest hue discrimination towards the long-wavelength (red) end of the spectrum (Stiles 1981), they can also respond to ultraviolet light, and there is no evidence that, for example, hummingbirds have greater spectral sensitivity or greater spectral discrimination ability in the red part of the spectrum (Goldsmith and Goldsmith 1979). Feeding experiments, where hummingbirds are given nectar in artificial flowers of different colours, show no inherited colour preferences; hummingbirds have temporary preferences that can be modified by conditioning (Proctor et al. 1996). So are there other clues as to how this mystery might be solved?

## The Visual System of Bees

One clue might come from the visual system of bees. Humans perceive light with a wavelength above approximately 600 nm as red (Buser and Imbert 1968). Most bees have three types of colour receptors, with sensitivity peaks at 340, 430, and 540 nm (Chittka 1996), although a very few bee species have sensitivity peaks at substantially longer wavelengths. For the majority, however, provided that the light source is sufficiently intense, red light (up to 650 nm) will stimulate the 540 nm receptor of bees (Chittka and Waser 1997). Bees will therefore perceive red objects. To discriminate red flowers from their green background, bees must rely essentially on the difference between the intensity of the signal that flower and foliage generate on the bees' ‘green’ (540 nm) receptor (Giurfa et al. 1996). Therefore, depending on the relative intensity of the green and red sources, bees may or may not be able to discriminate between red flowers and green foliage (Chittka and Waser 1997).

Because of the structure of their visual system, bees trained to feed at artificial red flowers take longer to find their goals than bees trained to feed at other-coloured flowers (Spaethe et al. 2001). In a real environment, where red flowers would be more camouflaged against the different shades and intensities of the green foliage, the ability of bees to discriminate red flowers should be further reduced.

## Colour Vision and Niche Partition

The fact that bees require more time to find red flowers than other-coloured flowers, together with some results from optimal-foraging theory, outlined here, could unlock the mystery and explain the association between red coloration and bird pollination in flowers.

When different animals, either from the same or different species, are forced to share some resources, any degree of specialization tends to result in habitat selection (Rosenzweig 1981). In 1992, Possingham, developed a ‘habitat selection’ model that showed how two nectar-feeding pollinator species, which differed in their foraging efficiency, would forage on two types of flowers. Although an abstract model, we can use it to illustrate how birds might interact with bees at different-coloured flowers.

Consider a community that includes bees and birds, and red and blue flowers. Let us assume that the flowers differ only in their colour, that there are only two patches of flowers (one of blue, the other of red flowers), and that the density of flowers is the same in both patches. (For a general analysis, with the same qualitative results, see Possingham 1992.)

The question is: how many birds and bees should forage at the red and blue patches so that their intake of nectar is maximised? The expected intake rate is the average amount of nectar obtained per flower (or standing crop) divided by the time it takes to find and exploit a flower. If the flowers are the same distance apart and birds can detect red and blue flowers equally well, then travel time is independent of flower colour. Under these circumstances, an ecological equilibrium, with birds exploiting red and blue flowers equally, would indicate that the amount of nectar available from both flower colours was identical.

Now add a few bees to this community of birds, sufficiently few that their intake of nectar is negligible. We know that the standing crop is the same at red and blue flowers. However, we also know that bees require more time to find red flowers than blue ones (Spaethe et al. 2001), so their intake rate of nectar will be higher at blue flowers, and they will all go to the blue patch.

If we continue to add bees one at a time to this community, then sooner or later, the number of bees will no longer be sufficiently low for us to ignore their depleting effect on the nectar available. What will happen at that point? Will bees now start visiting red flowers? Not yet. For a bee to visit the red patch, the difference in standing crop between red and blue flowers would have to be large enough to compensate for the difference in detection time. Before that happens, some birds will shift to the red patch. Indeed, since birds require the same time to detect red or blue flowers, some birds will move from the blue to the red patch as soon as bees start to noticeably reduce the nectar available from the blue flowers.

What Possingham's model predicts, therefore, is that when the number of bees is large enough, all birds will forage at the red patch. Only when the difference in standing crop between red and blue flowers is so large that it compensates for the reduced detectability of red flowers, will bees start visiting the red patch.

**Figure 3:**
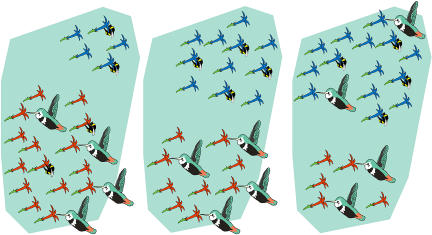
Possible Outcomes of Possingham's Model (1992) Each panel represents one possible outcome. If red flowers predominate (left), bees forage at blue and red flowers, while all birds forage at red flowers. If blue and red flowers are equally abundant (middle), there is complete resource partitioning, with bees foraging at blue flowers and birds at red flowers. If blue flowers become more abundant (right), all bees forage at blue flowers, while birds forage at red and blue flowers.

To conclude, there will be an association between red flowers and birds. Birds will exploit red flowers, and bees blue flowers. In addition, depending on the relative abundance of bees and birds (and of red and blue flowers), either birds or bees, but never both simultaneously, can also exploit the other flower type (Figure 3).

## Niche Partition and the Evolution of Red Flowers

Possingham's model (1992) helps to explain the ecological association between flower colour and pollinator type, provided that both flower colours and pollinator types are present—but why did the red colouration of these flowers evolve in the first place? We believe that the model can also help explain the evolution of red coloration in bird-pollinated flowers.

To understand the evolutionary process, consider a community where bees and birds are present, and where two flower species coexist. One flower type, the generalist flower, is blue and is efficiently pollinated by bees and birds alike. The blue Rocky Mountain penstemon, Penstemon strictus, provides a good example (Castellanos et al. 2003). The other flower type, or bird flower, is yellow and is efficiently pollinated by birds, but not by bees—the red beardlip penstemon P. barbatus provides an example of this type (Castellanos et al. 2003). If bee visits were costly for the ancestral bird flowers, they would experience a selective pressure to become red. Bees could impose several costs on the ancestral bird flowers; for example, the number of hummingbird visits may depend on the amount of nectar available in the flowers.

Throughout evolutionary history, there will be variability and heritability in flower colour (as documented for Mimulus by Bradshaw et al. 1995). Since both bees and birds easily detect and efficiently pollinate generalist blue flowers, there is no particular reason to expect that their colour will evolve in one direction or another. Things are otherwise for bird flowers, which are more efficiently pollinated by birds. For simplicity, consider that, at any given time, this bird flower comes in only two shades of colour, one of them with a slightly longer wavelength (an orange morph). On an ecological timescale, yellow flowers will be visited mainly by bees and orange flowers mainly by birds. Orange flowers, being more efficiently pollinated by birds, will therefore have higher fitness than yellow flowers, and given enough time, there will be selection for bird flowers to become orange. In the absence of other costs, mutant flowers with higher wavelengths (i.e., becoming redder) can invade a population of yellow flowers so long as bird flowers continue to be visited by bees (unpublished data). So bird flowers will continue to shift their colour until bees are completely excluded from the bird flowers or until further shifts deteriorate detectability by birds.

This explanation for the evolution of red coloration in bird-pollinated flowers differs from the one proposed by Raven (1972) in a key respect. In our view, the main point is not that bees fly over red flowers without seeing them; it is not even that they are unable to exploit red flowers efficiently in absolute terms. It is rather a question of relative efficiency that makes bees avoid red flowers when birds are depleting their nectar; it would work just as well if birds were colourblind and perceived red flowers as badly as flowers of other colours. Of course, Possingham's model (1992) is not incompatible with birds being more efficient than bees at exploiting red flowers, and the results would be strengthened if, as has been suggested (Raven 1972; Chittka and Waser 1997), birds are better at detecting red flowers than blue ones.

## Toward a Solution

Comparable problems can be found in other plant–pollinator systems. For example, when several species of bumblebees coexist, resource partitioning normally doesn't follow colour, but is dependent on different parameters: the corolla length of the plant and the proboscis length of the bee. Proboscis length affects the efficiency with which flowers of different depth are exploited (Inouye 1980); bumblebees with long proboscises preferentially exploit flowers with deep corollas, while bumblebees with short proboscises exploit shallow flowers (Heinrich 1976). But a bumblebee with a long proboscis can also exploit shallow flowers, and, to some extent, a bumblebee with a short proboscis can exploit deep flowers, if corollas are not too deep (although they will still leave some nectar behind). Indeed, when one bumblebee type is experimentally removed, the other one is seen to exploit both deep and shallow flowers (Inouye 1978). The same, we believe, should happen with flower colour: the experimental removal of birds should lead to the systematic exploitation of red flowers by bees, at least when corolla tube morphology does not prevent bees from accessing the nectar.

In fact, there is even no need to perform experimental bird removals, because plants provide us with a ready-made design: bees visit flowers searching for both nectar and pollen, while most birds exploit only the nectar. Hence, bees should readily collect pollen at red bird flowers. There are numerous examples of this, although in most cases they are indirectly documented. For example, solitary bees and syrphid and muscoid flies visit the red, hummingbird-pollinated flowers of Ipomopsis aggregata to collect pollen when hummingbirds visits are frequent, while bumblebee (Bombus appositus) visits to collect nectar are only common when hummingbird visits are rare (Mayfield et al. 2001). Outside the native range of bird-pollinated plants, the same phenomenon can be observed: in Spanish gardens, the honeybee collects pollen from Aloe arborescens plants. Bees cannot access the nectar, concealed at the bottom of the corolla tube. This is opportunistically collected by birds such as the Sardinian warbler Sylvia melanocephala (unpublished data).

Another comparison of interest concerns beetle-pollinated flowers, which in the Mediterranean region have open, bowl shapes and red coloration (Dafni et al. 1990). Amphicoma beetles are more efficient pollinators of these flowers than commonly occurring bees (Dafni et al. 1990), so the red coloration of these flowers might help to keep other visitors (possibly bees and flies) at bay. Indeed, other bowl-shaped flowers of different colours (such as yellow, white, and purple, e.g., in the genera Cistus and Helianthemum) are commonly visited by pollen-collecting bees and bumblebees. A particularly interesting test case is provided by the corn poppy Papaver rhoeas; in the eastern Mediterranean region, it is pollinated by beetles and does not reflect in the UV (Dafni et al. 1990), while in central and western Europe it reflects in the UV (Daumer 1958) and is pollinated by bees.

Although refinements of Possingham's model, such as developing a prey-model version, or introducing stochasticity or several foraging constraints, might help us determine the extent to which we should expect resource partitioning along the colour dimension to take place, it is, in our view, far more pressing to determine the extent and conditions under which bees exploit red flowers (i.e., through comparisons of pollen vs. nectar exploitation, bird exclusion experiments, etc.), the detection time of red flowers against a natural background, and the effect of flower colour and size on flight mode in the field. Only then will we be able to fully unravel the factors that solve this fascinating mystery.
